# The neuroendocrine immunomodulatory axis-like pathway mediated by circulating haemocytes in pacific oyster *Crassostrea gigas*

**DOI:** 10.1098/rsob.160289

**Published:** 2017-01-11

**Authors:** Zhaoqun Liu, Zhi Zhou, Qiufen Jiang, Lingling Wang, Qilin Yi, Limei Qiu, Linsheng Song

**Affiliations:** 1 Key Laboratory of Experimental Marine Biology, Institute of Oceanology, Chinese Academy of Sciences, Qingdao 266071, People's Republic of China; 2 Key Laboratory of Mariculture and Stock Enhancement in North China's Sea, Ministry of Agriculture, Dalian Ocean University, Dalian 116023, People's Republic of China; 3 University of Chinese Academy of Sciences, Beijing 100049, People's Republic of China

**Keywords:** *Crassostrea gigas*, neuroendocrine immunomodulatory axis, circulating haemocyte, membrane receptor, immune regulation

## Abstract

The neuroendocrine-immune (NEI) regulatory network is a complex system, which plays an indispensable role in the immunity of host. In this study, a neuroendocrine immunomodulatory axis (NIA)-like pathway mediated by the nervous system and haemocytes was characterized in the oyster *Crassostrea gigas*. Once invaded pathogen was recognized by the host, the nervous system would temporally release neurotransmitters to modulate the immune response. Instead of acting passively, oyster haemocytes were able to mediate neuronal immunomodulation promptly by controlling the expression of specific neurotransmitter receptors on cell surface and modulating their binding sensitivities, thus regulating intracellular concentration of Ca^2+^. This neural immunomodulation mediated by the nervous system and haemocytes could influence cellular immunity in oyster by affecting mRNA expression level of TNF genes, and humoral immunity by affecting the activities of key immune-related enzymes. In summary, though simple in structure, the ‘nervous-haemocyte’ NIA-like pathway regulates both cellular and humoral immunity in oyster, meaning a world to the effective immune regulation of the NEI network.

## 1. Introduction

A neuroendocrine-immune regulatory (NEI) network is proposed to bring the self-regulated immune system into conformity with other body systems. This hypothesis is based on the existence of reciprocal interactions between immune and neuroendocrine systems in the host [1]. In this network, the nervous system regulates immune responses through humoral and neuronal routes [2] by activating the immune system to synthesize cytokines and immune-related enzymes to modulate immune responses [3,4]. In humans, the nervous system-mediated immunity is regulated through systemic, regional and local routes using the NEI regulatory axes (NIAs) as a structural base [5]. The peripheral nervous system (PNS) serves as the first line defending at local sites of inflammation by releasing neuropeptides that generally increase local inflammatory responses. The sympathetic (or adrenergic) nervous system (SNS) and the parasympathetic (or cholinergic) nervous system (PNS) generally inhibit inflammation at a regional level by innervating immune organs [2,3,6]. Neuroendocrine responses controls inflammation at a systemic level through the hypothalamic–pituitary–adrenal (HPA), hypothalamic–pituitary–gonadal (HPG) and hypothalamic–pituitary–thyroid hormone (HPT) axes, in which the adrenal cortex, ovaries and testes, and thyroid gland release glucocorticoids, sex hormones and thyroid hormones, respectively [2,7,8]. However, the structure of the nervous system is relatively simple in invertebrates, while the diversity and complexity increases along with the evolution [9,10]. For example, the neurons in Cnidaria interact with each other within a reticular nervous system [11], while in Platyhelminthes, the neurons form a trapezoidal nervous system, indicating a dramatic progress in evolution [12]. Although the structural complexity of nervous system is different among phyla, a great degree of similarity in endocrine and immune systems has been revealed, and analogous NIAs are characterized in invertebrates [3]. In cnidarians, endocrine cells occur as scattered neurons and epithelial cells in the epidermis and gastrodermis [13]. The neuropeptides released by neuroendocrine cells act as transmitters to mediate neuronal communications within the nerve net and to stimulate effector organs [14,15]. Similarly, the neurosecretory cells (NSCs) and neurohemal structures located in the glial sheath of the nervous system have been described in several mollusc species [16]. In terrestrial snails (pulmonates), several peptide hormones producing ‘nuclei’ have been described within the brain cortex [17]. Because the nervous system in invertebrates is far more primitive than that in higher forms of life, the endocrine and immune systems, particularly the NSCs and immune cells, become extremely important for the functional integrity of NEI network.

Haemocytes in invertebrates are regarded as main immune cells [18]. The haemocytes play a key role in digestion, metabolite transport, shell repair and especially in immune responses [19–21], including phagocytosis [4], synthesizing and releasing cytokines and immune-related enzymes [22–24], as well as producing reactive oxygen intermediates [25]. In marine molluscs, classical hormones and neurotransmitters released by the neuroendocrine system can bind specific receptors on haemocytes to modulate immune activity [3]. For example, muscarinic acetylcholine receptors on the surface of oyster *Crassostrea gigas* haemocytes regulate TNF expression and apoptosis process [4]. Delta opioid receptors for molluscan [Met^5^]-enkephalin modulate the phagocytic and antibacterial activities of haemocytes through the second messengers Ca^2+^ and cAMP [26]. This ‘nervous-haemocyte’ structure in marine invertebrates can be considered as an NIA-like pathway to regulate numerous immunological processes.

Invertebrates lack the complexity of adaptive immunity and rely solely on innate immunity with mediation of both cellular and humoral components [27]. The invertebrate innate immune responses are largely under the regulation of NEI network, mainly through the ‘nervous-immunocytes’ NIA-like pathway [28]. For example, the treatments with neurotransmitters including acetylcholine (ACh), norepinephrine (NE) and [Met^5^]-enkephalin (ENK) induced the phagocytosis [3] and apoptosis of haemocytes, and the production of cytokines (such as IL-17, TNF-α and IFN-γ) in molluscs [22–24]. In addition, ACh and high concentration of NE binding to their specific receptors significantly repressed the bacteria-induced immune responses [21,29–31]. NE exerted various effects on nitric oxide synthase (NOS) activities and NO production at different immune stages via a novel α/β-adrenoceptor-cAMP/Ca^2+^ regulatory pattern [32–34]. These findings further suggested that the ‘nervous-haemocyte’ NIA-like pathway based on the binding of neurotransmitters or hormones to their receptors on haemocyte surface might be far more important for the invertebrates than we have learned.

Molluscs, such as the oyster *C. gigas*, have evolved a primitive neuroendocrine system and a sophisticated immune system [3]. Their haemocytes are able to recognize a variety of stimuli and set up correspondingly complex responses [27], making them perfect models for the study of NEI network. Recently, a debate has been raised as to whether the haemocytes mediate neuroendocrinal regulation in the first place, or just passively relay signals from neuroendocrine to the downstream pathways. Study on the immunomodulation patterns of haemocytes under neuroendocrinal regulation will pave a new way for the better understanding of functions, origin and evolution of NEI network in invertebrates. The purposes of this study are to (i) explore the regulation pattern of oyster haemocytes under certain kind of neurotransmitter, (ii) draw the regulation pattern of oyster haemocytes under multi-kind of neurotransmitters and (iii) evaluate the immunological significance of the haemocyte-mediated neuroendocrine immunomodulation for innate immunity in marine molluscs.

## 2. Material and methods

### 2.1. Oysters and treatments

Oysters *C. gigas* (averaging 150 mm in shell height) were collected from a local farm in Qingdao, Shandong Province, China, and maintained in aerated seawater at 18°C for two weeks before processing. The food of powdered algae (commercially purchased) was added to the water every other day. The seawater in the aquaria was replaced every day.

For the lipopolysaccharide (LPS) stimulation experiment on adult, 100 µl of LPS (0.5 mg ml^−1^ in seawater) was injected into the adductor muscle of each oyster in the LPS group, while the same volume of seawater was injected into each oyster in the control (SW) group. Treatments of oyster haemocytes with neurotransmitters or neurotransmitter receptor antagonists were conducted according to our previous report [4]. NE at a low final concentration of 0.25 µM (Sigma Aldrich), ENK (15 nM, Sigma Aldrich), and a mixture of NE (0.45 µM) and ENK (25 nM) was added to the cell culture medium, respectively and synergistically. In the experiment of antagonist incubation, CgA1AR-1 (NE receptor) specific antagonist Doxazosin mesylate (DOX, TOCRIS) at a final concentration of 10.0 µmol l^−1^ (paper not published) and CgDOR (ENK receptor) specific antagonist 7-benzylidenenaltrexone maleate (BNTX, TOCRIS) at a final concentration of 10.0 µmol l^−1^ [26] were added to the cell culture medium, respectively, and synergistically 3 h ahead of neurotransmitter treatment. Meanwhile, 100 µl of 1.0 mmol l^−1^ DOX and BNTX was also injected into each adult oyster 6 h before LPS stimulation in the adult oyster experiments. Specifically, Mix 1 referred to mixture of antagonists DOX and BNTX, Mix 2 referred to mixture of high concentration of NE (0.45 µM) and ENK (25 nM). Haemocytes or individuals receiving seawater treatments instead of neurotransmitter or antagonist incubation were employed as the control group.

### 2.2. Sample collection and haemocyte primary culture

Oyster haemolymph was aspirated from the blood sinus with a thin syringe, and centrifuged at 800*g* to harvest the haemocytes and serum. Haemolymph from five oysters was pooled together as one duplicate. Three parallel duplicates were employed for each test. Trizol reagent (1 ml, Invitrogen, USA) was added to each tube containing haemocytes, and these samples were frozen immediately with liquid nitrogen and stored at −80°C for the subsequent RNA extraction and enzyme activity determination.

Primary cell culture was carried out according to the protocol of Jiang *et al.* [34]. Haemocytes collected from haemolymph were resuspended in modified Leibovitz-15 medium (Gibco, Life Technology) supplemented with 345.7 mmol l^−1^ NaCl, 7.2 mmol l^−1^ KCl, 5.4 mmol l^−1^ CaCl_2_, 9.0 mmol l^−1^ MgSO_4_, 41.0 mmol l^−1^ MgCl_2_, 115.5 mmol l^−1^ glucose, 10% fetal bovine serum (FBS), 299.1 µmol l^−1^ penicillin G, 171.9 µmol l^−1^ streptomycin, 83.8 µmol l^−1^ gentamicin and 0.11 µmol l^−1^ amphotericin B (pH 7.4). The resuspended haemocytes were adjusted to a cell count of 1 × 10^6^ cells ml^−1^, and cultured in a 24-well plate (Costar). The cell viability was detected by Trypan Blue exclusion technique using commercial kit (Beyotime Biotechnology).

### 2.3. RNA extraction and quantitative real-time PCR

Total RNA was isolated from the oyster haemocytes using Trizol reagent according to its protocol. The synthesis of the first-strand cDNA was carried out based on Promega M-MLV RT Usage information using the DNase I (Promega)-treated total RNA as template and oligo(dT)-adaptor as primer. The synthesis reaction was performed at 42°C for 1 h, terminated by heating at 95°C for 5 min. The cDNA mix was diluted to 1 : 50 and stored at −80°C.

SYBR green quantitative real-time PCR was employed to evaluate the mRNA expression level of three oyster TNF genes (CGI_10005109, CGI_10005110 and CGI_10006440) using the 2^−ΔΔ*C*^_t_ method according to Zhou *et al.* [35]. All the primers used are listed in table 1 and the fragment (168 bp) of oyster elongation factor (CgEF, CGI_10012474) was detected as endogenous control. Six duplicates were tested for each sample.

**Table 1.** Sequences of the primers used in the experiment.

primer	sequence (5′–3′)	sequence information
P1 (forward)	CGCAATGGTCGCTTGGTGGTC	real-time TNF (CGI_10005109) primer
P2 (reverse)	CGTAGGGGCGGAAGGTCTCG	real-time TNF (CGI_10005109) primer
P3 (forward)	CAACGGTCTAACTTACCATCCAAAC	real-time TNF (CGI_10005110) primer
P4 (reverse)	TGGTGGTAGATAAAATGGGACAGTG	real-time TNF (CGI_10005110) primer
P5 (forward)	ATTGGAGCACCTGGAGGATAAG	real-time TNF (CGI_10006440) primer
P6 (reverse)	CAGTCTTCCGTGCTGGTATTTC	real-time TNF (CGI_10006440) primer
P7 (forward)	ATCCTTCCTCCATCTCGTCCT	real-time CgEF (CGI_10012474) primer
P8 (reverse)	GGCACAGTTCCAATACCTCCA	real-time CgEF (CGI_10012474) primer
M13-47	CGCCAGGGTTTTCCCAGTCACGAC	pMD18-T simple vector primer
RV-M	GAGCGGATAACAATTTCACACAGG	pMD18-T simple vector primer
P9 (forward)	GGCCACGCGTCGACTAGTACT_17_	oligo(dT)-adaptor

### 2.4. Quantification of NE and ENK in oyster haemolymph

The temporal concentration changes of NE and ENK in oyster haemolymph after LPS stimulation were determined by norepinephrine ELISA kit (Abnova) and Met-enkephalin ELISA kit (MyBioSouce) [34,36]. Briefly, NE was extracted from samples using a cis-diol-specific affinity gel, acylated and then derivatized enzymatically. After the samples were equilibrated, free NE and free NE-antibody complexes were removed by three rounds of washing with wash buffer. The antibody bound to the solid phase was detected by using an anti-rabbit IgG-peroxidase conjugated with TMB as a substrate. Similarly, serum for ENK determination was incubated with MENK-HRP conjugated in pre-coated plate for 1 h. After five rounds of washing with wash buffer, the wells were then incubated with a substrate for HRP enzyme. The product of the enzyme-substrate reaction forms a blue-coloured complex. Finally, a stop solution was added to stop the reaction, which would then turn the solution yellow. Both reactions were monitored by a microtitre plate reader (BioTek, USA) at 450 nm. Quantification of samples was achieved by comparing their absorbance with a reference curve (*n* = 6).

### 2.5. Determination of intracellular Ca^2+^

Ca^2+^ was detected with Fluo-3 AM fluorescent probe (Beyotime, 1.0 µmol l^−1^) according to the protocol. The haemocytes were incubated with Fluo-3 AM fluorescent probe (5.0 µl per well) at 37°C for 45 min to allow the Fluo-3 AM turning into Fluo-3 completely in the cells. The cells were then washed twice with 1× PBS (Gibco, pH 7.4) and digested with trypsin (Beyotime). After a centrifugation at 1000*g* for 5 min, the fluorescence intensity of the cells was determined by flow cytometry (BD FACS Aria II SORP).

### 2.6. Measurement of key immune-related enzymes

The activities of three immune-related enzymes including superoxide dismutase (SOD), catalase (CAT) and lysozyme (LYZ) in the oyster haemolymph were measured by the kits (Nanjing, Jiancheng, A001-1, A007 and A050-1) according to the protocol. Total SOD activity was determined by the hydroxylamine method. One SOD activity unit was defined as the enzyme amount causing 50% inhibition in 1 ml reaction solution. Total CAT activity was determined by using spectrophotometry to measure yellowish complex compound yielded from the reaction between hydrogen peroxide and ammonium molybdate [37]. As for the detection of LYZ activity, 20 microlitre haemolymph supernatant was added to 200 ml bacteria suspension (0.25 mg ml^−1^ in bacterial buffer supplied from the kit). The mixture was incubated at 37°C for 15 min, and then bathed in ice for 3 min. The reduction of absorbance at 530 nm was measured at room temperature using a microtitre plate reader (BioTek, USA). The total LYZ activity was measured by comparing the turbidimetry of the samples with that of LYZ standard solution (2.5 mg ml^−1^, supplied by the kit) [32].

### 2.7. Statistical analysis

All data were given as means ± s.d. and subjected to one-way analysis of variance (one-way ANOVA) followed by a multiple comparison (S-N-K). Differences were considered significant at *p* < 0.05.

## 3. Results

### 3.1. Temporal changes of NE and ENK concentrations and their receptor mRNA transcripts after LPS stimulation

**Figure 1.**
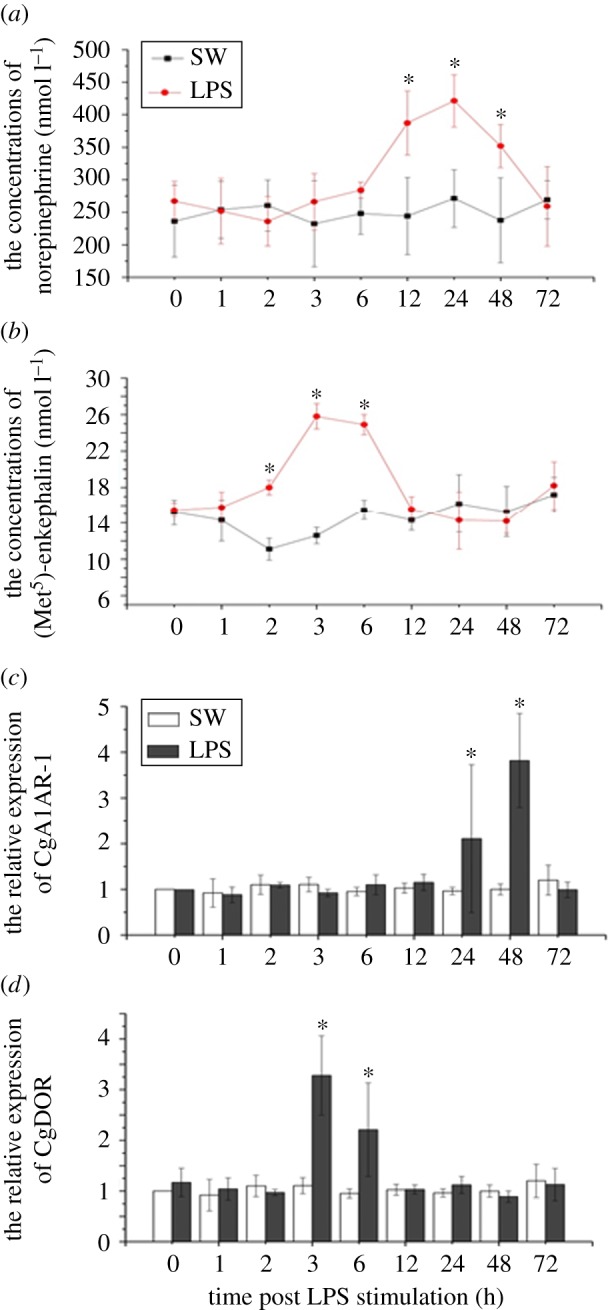
Determination of (*a*) norepinephrine (NE) and (*b*) [Met^5^]-enkephalin (ENK) concentrations in serum, and the mRNA expression level of (*c*) CgA1AR-1 and (*d*) CgDOR in haemocyte after LPS stimulation.

The concentrations of both NE and ENK in oyster haemolymph increased significantly after LPS stimulation at different time points (figure 1*a*,*b*). ENK content began to increase at 2 h post-injection and peaked at 3 h (*p* < 0.05) compared with that in the SW group. After 12 h of stimulation, ENK concentration decreased to the initial level (*p* > 0.05). Unlike ENK, NE concentration increased at late stages during the immune response. From 12 to 48 h, NE concentration in haemolymph was higher than that in the control group (*p* < 0.05), while no significant difference was observed during the early stages of the immune response (*p* > 0.05).

Interestingly, the changes of the expression levels of NE and ENK receptors CgA1AR-1 and CgDOR were consistent with their ligands after *in vivo* LPS stimulation (figure 1*c*,*d*). The mRNA transcript expression of CgA1AR-1 increased to a significant level at 24 and 48 h (late stages of the immune response) after LPS injection (*p* < 0.05), which conformed with the content fluctuation of NE. The expression of CgDOR mRNA transcript exhibited similar change trends with ENK concentration, reaching the highest level at 3 h and 6 h (early stages of the immune response) post-LPS stimulation (*p* < 0.05).

### 3.2. Alterations in intracellular Ca^2+^ contents after treatments of NE, ENK and antagonists of their receptors in primarily cultured haemocytes

**Figure 2.**
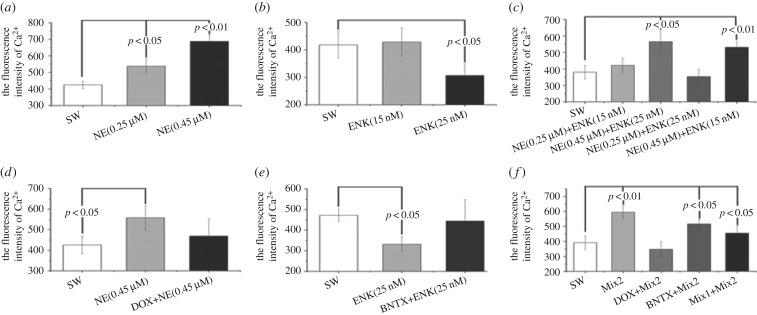
Measurement of intracellular Ca^2+^ *in vitro* after antagonists incubation and neurotransmitters treatment on primarily cultured haemocyte.

The content of intracellular second messenger Ca^2+^ in primarily cultured haemocytes after treatments of NE, ENK, DOX and BNTX was determined with flow cytometry (figure 2). Both low (0.25 µM) and high (0.45 µM) concentrations of NE could induce a significant increase in intracellular Ca^2+^ content (figure 2*a*, *p* < 0.05), while blockade of NE receptor CgA1AR-1 with antagonist DOX reverted such changes (figure 1*d*). Oppositely, high concentration (25 nM) of ENK downregulated intracellular Ca^2+^ level (figure 2*b*, *p* < 0.05), which was reverted by inhibiting ENK receptor CgDOR with its antagonist BNTX (figure 2*e*).

When haemocytes received synergistic treatments of NE and ENK, the changes of Ca^2+^ content were basically similar to those in the NE treatment group (figure 2*c*). The concentrations of intracellular Ca^2+^ in the NE (0.45 µM) + ENK (15 nM) group and the NE (0.45 µM) + ENK (25 nM) group were much higher than that in the SW group (*p* < 0.05), while no significant content changes were detected in either the NE (0.25 μM) + ENK (25 nM) group or the NE (0.25 µM)+ENK (15 nM) group (*p* > 0.05). Moreover, Ca^2+^ contents increased dramatically in the Mix2 (NE + ENK), BNTX + Mix2 and Mix1 (DOX + BNTX) + Mix2 groups (*p* < 0.05), while no significant change was observed in the DOX + Mix2 group (*p* > 0.05) in comparison with SW group.

### 3.3. The change of intracellular Ca^2+^ contents in oyster haemocytes after receptor antagonism and LPS stimulation

**Figure 3.**
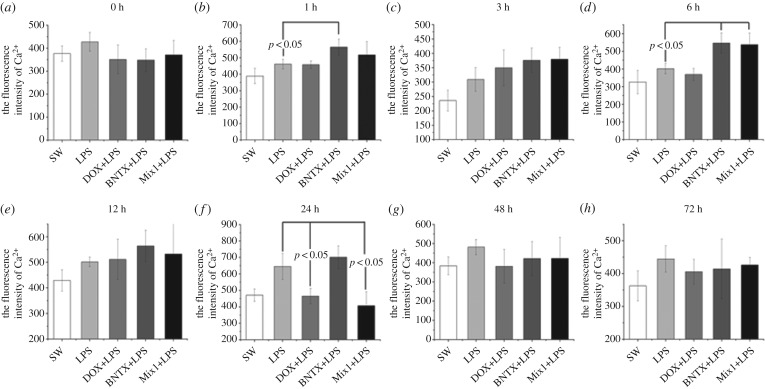
Detection of intracellular Ca^2+^ in haemocytes after *in vivo* antagonists incubation and LPS stimulation on oysters.

Haemocyte Ca^2+^ concentration was detected after *in vivo* treatments of LPS and the antagonists of NE and ENK receptors (figure 3). No significant change in intracellular Ca^2+^ concentration was detected in all groups at 3 h, 12 h, 48 h and 72 h after LPS stimulation. At 1 h after LPS stimulation, intracellular Ca^2+^ contents increased significantly in the BNTX + LPS group (*p* < 0.05) compared with that in the LPS group. Ca^2+^ concentration in haemocyte at 6 h increased dramatically in the BNTX + LPS and Mix1 + LPS groups (*p* < 0.05), while the concentration remained unchanged in the other experimental groups (*p* > 0.05). Also, at 24 h post-LPS stimulation, Ca^2+^ contents in the DOX + LPS and Mix1 + LPS groups decreased remarkably compared with the LPS group (*p* > 0.05).

### 3.4. Expression of oyster TNF mRNA transcripts in oyster haemocytes after receptor blockade and LPS stimulation

**Figure 4.**
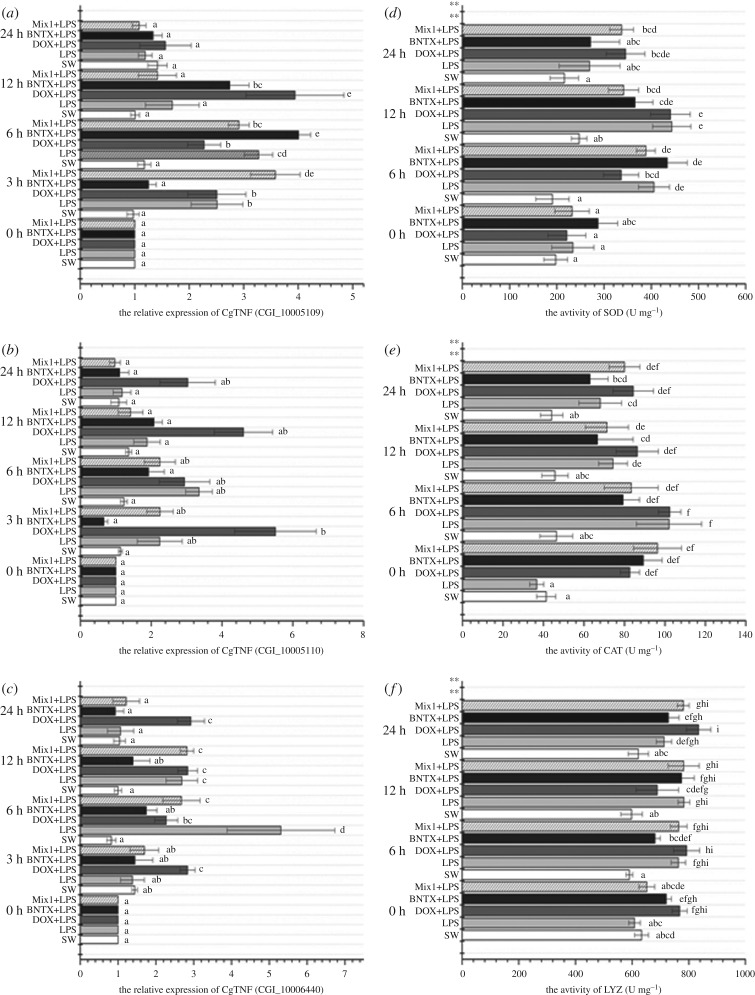
Determination of mRNA expression level of oyster TNF in haemocyte and the activities of key immune-related enzymes in serum after antagonists incubation and LPS stimulation.

The mRNA expression levels of three oyster TNFs (CGI_10005109, CGI_10005110 and CGI_10006440) were examined with quantitative real-time PCR (figure 4*a*–*c*). All their expressions increased significantly in response to LPS stimulation. In general, the expression of CGI_10005109 peaked at 3 h in the Mix1 + LPS group, at 6 h in the BNTX + LPS group and at 12 h in the DOX + LPS group, in comparison with that in the SW group (figure 4*a*, *p* < 0.05). The expression level of CGI_10005110 was relatively high in the DOX + LPS group at both 3 h and 12 h post-LPS stimulation (figure 4*b*, *p* < 0.05). Also, CGI_10006440 was basically overexpressed in the DOX + LPS group from 3 h to 24 h post-LPS challenge (figure 4*c*, *p* < 0.05).

### 3.5. Temporal changes in immune-related enzyme activities after treatments of receptor antagonists and LPS stimulation

The activities of three key immune enzymes, SOD, CAT and LYZ, were determined after *in vivo* LPS stimulation the antagonists treatments of NE/ENK receptors (figure 4*d*–*f*). LPS stimulation remarkably increased the activities of SOD, CAT and LYZ. After antagonists inhibition treatments, LYZ and CAT activities increased significantly in all groups from 6 h to 24 h (figure 4*e*,*f*; *p* < 0.05), while SOD activities were much higher at 6 h in the BNTX + LPS group and at 12 h in the DOX + LPS group post-LPS stimulation (figure 4*d*; *p* < 0.05), compared with that in the control group.

## 4. Discussion

Communication between the neuroendocrine and immune systems is of great importance for the maintenance of homeostasis and body integrity in both vertebrates and invertebrates [38,39]. As a structural basis for neural regulation, a complicated neuroendocrine immunomodulatory axis (NIA) exists in vertebrate animals [40]. However, some lower forms of life, such as invertebrates, lack endocrine and immune organs, and harbour a primitive nervous system. It is a challenging question that such a simple NEI network could conduct complex functions similar to those in vertebrates. In a previous study, we have demonstrated that a simple-in-structure NEI network does exist in marine molluscs to mediate a vast array of immunomodulatory processes including immunocytes phagocytosis, apoptosis and production of cytokines [3]. In this study, we further explored the underlying mechanisms by which circulating haemocytes mediated the nervous-immune regulation pattern in pacific oyster *C. gigas*, hoping to better understand the NEI network in marine molluscs.

It is interesting that the concentrations of NE and ENK, two crucial neurotransmitters for immune regulation in oyster [41,42], changed temporally after LPS stimulation compared with those in the control group (figure 1*a*,*b*). The NE production increased significantly at late stages of the immune response (from 12 h to 48 h), while the ENK production increased significantly at early stages (from 2 h and 6 h). In the meantime, the mRNA expression levels of NE receptor CgA1AR-1 and ENK receptor CgDOR in haemocytes were also changed consistently with their ligands after LPS stimulation (figure 1*c*,*d*). NE belongs to catecholamines (CA), which are long-lasting neurotransmitters released by sympathetic nervous system and basically inhibit the increase in immune response level in vertebrates [43,44]. In molluscs, NE was able to modulate both haemocyte ROS level and phagocytosis in oyster *C. gigas* via beta-adrenergic receptors [45,46]. On the contrary, ENK generally upregulates immune response by influencing haemocyte phagocytosis, apoptosis and haemolymph antibacterial activities in molluscs [36,41]. Also, neurotransmitters, like hormones or neuromodulators, are able to modulate a vast array of physiological activities at an extremely low concentrations [47]. However, this elaborate regulatory network can be disrupted when the synthesis and release of neurotransmitters are in disorder [2,48], which means even a slight change in concentrations could entail a completely different result. This mechanism works for both vertebrates and invertebrates. Therefore, in this study, we determined the endogenous contents of ENK and ENK before the neurotransmitter treatment, and proper neurotransmitter concentrations were employed. According to the results, the temporal change in NE and ENK concentrations in oyster haemolymph post-LPS stimulation indicated that the oyster could carry out precise NEI regulation by releasing different neurotransmitters or hormones at different stages during the immune response, and thus could properly modulate the immune system through NEI network. Moreover, the expression alterations in CgA1AR-1 and CgDOR transcripts implied that oyster haemocytes mediated neuroendocrine immunomodulation in the first place during the immune response by controlling the mRNA expression of neurotransmitter or hormone receptors, instead of being passively regulated by neuroendocrine signals. Therefore, we hypothesize that there is an NIA (neuroendocrine immunomodulation regulatory axis)-like pathway in oyster to mediate nervous-immune regulation mainly by circulating haemocytes.

The regulation patterns of NE and ENK on primarily cultured haemocytes *in vitro* were further explored to validate the NIA-like pathway in oyster. Because intracellular Ca^2+^ has been used to reflect the binding of NE and ENK to their receptors and the activation of downstream immune-related pathways [21,26], the Ca^2+^ contents in primarily cultured haemocytes were determined in this study to illustrate how haemocytes mediated NE and ENK regulation. A high concentration of NE induced the accumulation of intracellular Ca^2+^, while a high concentration of ENK triggered the decrease of Ca^2+^ contents. When haemocytes received synergistic modulation of NE and ENK, Ca^2+^ concentration increased significantly, like that in NE treatment groups. These results suggested that there was a primitive synergistic NEI regulatory network in oyster, in which the immune cells were more sensitive to NE than ENK.

Neurotransmitters or hormones accomplish their functions by binding to their specific receptors on the surface of immune cells [4]. It has been reported that oyster haemocytes could express receptors for several neurotransmitters, including NE, ACh, ENK, serotonin (5-HT) and γ-aminobutyric (GABA) [21,26,31,36,49]. Blockade of these receptors with specific antagonists could significantly inhibit the regulation of neurotransmitters [4,21,26,31,49]. In this study, receptors on haemocytes were inhibited by antagonists both *in vivo* and *in vitro* to evaluate whether circulating haemocytes mediate neural immunomodulation by these transmembrane receptors. The increase of intracellular Ca^2+^ concentration caused by NE could be reverted after the treatment of CgA1AR-1 antagonist DOX. Similarly, the blockade of ENK specific receptor CgDOR with antagonist BNTX could stop the decrease of Ca^2+^ content induced by ENK incubation. Furthermore, inhibition of both CgA1AR-1 and CgDOR with DOX and BNTX significantly affected the immunomodulation of NE and ENK, while nearly no difference could be observed once CgDOR instead of CgA1AR-1 was blocked. These results collectively indicate that the oyster neuroendocrine system mediated immunomodulation mainly through the neurotransmitter receptors on cell surface. The NEI network in the oyster is more sensitive to NE than ENK under NE/ENK synergistic regulation pattern, possibly due to the mediation of transmembrane receptors.

The neural immune regulation is a long-lasting process, involving various neurotransmitters, hormones and neuropeptides [50]. Oysters possess only primitive nervous system and immune-related tissues and cells. They do not have a sophisticated nervous system or endocrine and immune organs to conduct elaborate physiological regulation. However, oysters survive well and are distributed all over the world, suggesting that they have evolved perfect homeostasis-maintaining systems including the NEI network to deal with the harsh environments. In this study, the temporal change in intracellular Ca^2+^ concentration after LPS stimulation, and the involvement of CgA1AR-1 and CgDOR in the process were explored in order to find some evidence for the existence of a simple but ‘smart’ NEI network in oyster. After *in vivo* LPS stimulation on oysters, the intracellular Ca^2+^ in haemocytes in the BNTX + LPS group increased significantly at 1 h and 6 h compared with that in the control and DOX + LPS groups, suggesting that ENK modulation on haemocyte immune response might dominate during early stages (0–6 h). Previous studies have demonstrated that ENK is capable of upregulating the immune response level under pathogen challenge in oysters and scallops [26,36,41]. Here, ENK modulation pattern in response to LPS treatment indicated that oysters could respond to stimuli in a very short time and release neurotransmitters such as ENK to enhance the immune response and eliminate the invading pathogens immediately. Moreover, Ca^2+^ concentrations decreased significantly in DOX + LPS and Mix1 + LPS groups, compared with that in control and BNTX + LPS groups at 24 h post-LPS stimulation, suggesting that during late stages of the immune responses (24–48 h), NE instead of ENK might play predominant role during the haemocyte immune response. NE basically could downregulate the immune response level caused by various stimulations in molluscs [3,21]. In this study, NE decreased the immune response in order to avoid the damage caused by excessive inflammation, maintaining body homeostasis. In summary, all these results demonstrated that the NEI network in oyster with simple structure was far more complex than we had realized. Haemocytes played an important role in this network by initially mediating the regulation of various neurotransmitters at different stages of the immune response, effectively eliminating invading pathogens and maintaining body homeostasis at the same time.

Neurotransmitters regulate the immune response by both cellular and humoral pathways [51,52], in which cytokines such as TNF, IL and IFN, and immune-related enzymes such as SOD, CAT and LYZ, are of great importance [22–24,35,53,54]. In this study, LPS stimulation induced the expression of all the three oyster TNF mRNA transcripts (CGI_10005109, CGI_10005110 and CGI_10006440) and the enzyme activities of SOD, CAT and LYZ (figure 4). It was reported that ENK treatment induced the downregulation of CGI_10005109 expression and the upregulation of CGI_10005110 and CGI_10006440 expression at mRNA level, while NE treatment decreased the mRNA expression of all these three TNF genes [3]. In this study, the expression of CGI_10005109 peaked in the BNTX + LPS group at 6 h and in the DOX + LPS group at 12 h, implying that ENK regulation was in predominance during the early stage of the immune response, while NE regulation was in predominance during the late stage of the immune response. Furthermore, the expression level of CGI_10005110 was relatively high in the DOX + LPS group at both 3 h and 12 h post LPS stimulation, while CGI_10006440 was basically overexpressed in the DOX + LPS group from 3 h to 24 h post LPS challenge, suggesting that NE regulation was more obvious than ENK regulation. Based on these results, we believe that ENK immunomodulation holds predominant position during early stage of the immune response, while NE immunomodulation dominates during late stage of the immune response. Similar regulation patterns were also observed in the determination of enzyme activities. After antagonists incubation, LYZ and CAT activities increased significantly in nearly all groups from 6 h to 24 h, and SOD activities were much higher in the BNTX + LPS group at 6 h and in the DOX + LPS group at 12 h post-LPS stimulation. These results once again support the conclusion that haemocytes initially mediated the neuronal immunomodulation with receptors on cell membrane, composing a simple-in-structure by smart-in-function NIA-like pathway in oyster.

**Figure 5.**
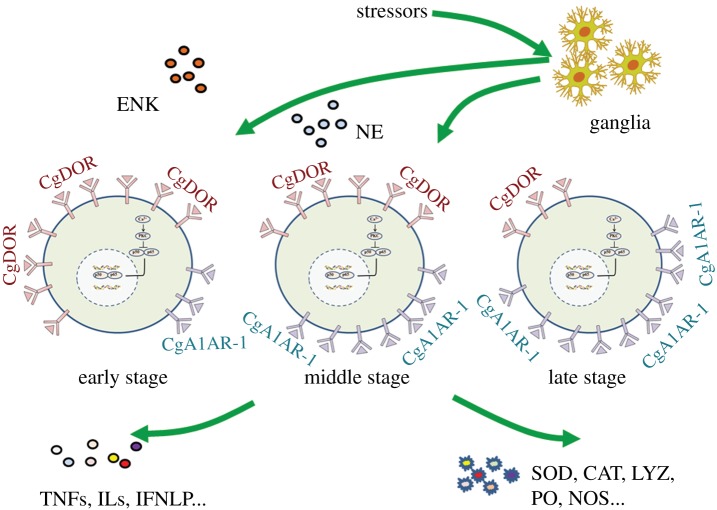
The ‘nervous-haemocyte’ NIA-like pathway in oyster.

Activation of the innate immunity in response to pathogen provides signals for the activation of the neuroendocrine system to synthesize and release neurotransmitters, hormones and neuropeptides to terminate inflammation. Though lacking a sophisticated nervous system and endocrine/immune organs, the oyster has evolved a simple-in-structure by smart-in-function NEI network, in which haemocytes play central roles. The oyster NEI network initially mediated the neuronal immunomodulation during the immune process to regulate both cellular and humoral immunity (figure 5) through a nervous-haemocyte NIA-like pathway. For the first time, the invertebrate NEI network has been illustrated in oyster, and this network is supposed to be as exquisite and effective as that in vertebrates.

## Data accessibility

Datasets are available in Dryad Data Repository: http://dx.doi.org/10.5061/dryad.7d6c5 [55].
